# Loneliness during COVID-19 and its association with eating habits and 24-hour movement behaviours in a sample of Canadian adolescents

**DOI:** 10.1016/j.pmedr.2023.102287

**Published:** 2023-06-16

**Authors:** Saniya Tandon, Karen A. Patte, Gary S. Goldfield, Scott T. Leatherdale, Jean-Philippe Chaput

**Affiliations:** a School of Epidemiology and Public Health, University of Ottawa, Ottawa, Ontario, Canada; b Department of Health Sciences, Faculty of Applied Health Sciences, Brock University, Niagara Region, St. Catharines, Ontario, Canada; c Healthy Active Living and Obesity Research Group, Children’s Hospital of Eastern Ontario Research Institute, Ottawa, Ontario, Canada; d Department of Pediatrics, Faculty of Medicine, University of Ottawa, Ottawa, Ontario, Canada; e School of Public Health Sciences, University of Waterloo, Waterloo, Ontario, Canada

**Keywords:** Isolation, Physical activity, Screen time, Sleep duration, Breakfast consumption, Teenagers, Coronavirus, COMPASS study

## Abstract

Loneliness, a feeling of distress, has aggravated due to the COVID-19 pandemic lockdowns and reduced social interactions. The objective of this study was to explore whether increased loneliness due to the COVID-19 pandemic was associated with various health behaviours in adolescence, a critical period for the development of lasting lifestyle habits. We used self-reported data from 40,521 Canadian adolescents aged 12–19 years (collected between November 2020 and June 2021) for this cross-sectional study. Logistic regression was used to predict the odds of skipping breakfast and not meeting movement behaviour guidelines [moderate-to-vigorous physical activity (≥60 min/day), recreational screen time (≤2 h/day), sleep duration (≥8 h/day)] among adolescents with increased loneliness due to the COVID-19 pandemic. We found higher odds of skipping breakfast [boys: OR 1.40 (95% CI: 1.32, 1.49), girls: OR 1.62 (95% CI: 1.53, 1.71)], exceeding screen time guidelines [boys: OR 1.43 (95% CI: 1.24, 1.66), girls: OR 1.72 (95% CI: 1.54, 1.92)], and insufficient sleep duration [boys: OR 1.38 (95% CI: 1.28, 1.48), girls: OR 1.36 (95% CI: 1.27, 1.45)] in adolescents with increased loneliness (versus decreased/stayed the same loneliness group). However, we found clinically insignificant results with moderate-to-vigorous physical activity. Future longitudinal studies in adolescents are needed to confirm the directionality of these associations. Recovery efforts are needed to support adolescent social health and establish healthy behavioural habits across the lifespan.

## 1 Introduction

Loneliness is defined as a feeling of distress that can arise from individuals’ perceived inadequacy of the quantity and quality of their social relationships (e.g., lack of support, low-quality friendships, having limited social contacts) (Perlman and Peplau, 1981). Loneliness is a significant risk factor for developing various physical and mental health conditions (Christiansen et al., 2021), and evidence indicates a gradual increase in levels of loneliness across adolescence and young adulthood (MacDonald et al., 2022).

Adolescence is a period of significant transition and is associated with increased vulnerability to risk-taking and impulsive behaviour, initiation of intimate relationships, greater autonomy from parental/family figures, figuring out their identity, and heightened importance of peer acceptance (Spear and Kulbok, 2004). Public health measures used to tackle the ongoing COVID-19 pandemic have affected the daily routines of adolescents. Decreased social interactions with peers and conflicts with parents/guardians/family members may lead to reduced communication and emotional support, enhancing feelings of loneliness in adolescents (Heinrich and Gullone, 2006).

Loneliness is a recognized public health concern and has exacerbated during the COVID-19 pandemic due to the lockdowns and decreased social interactions (Ernst et al., 2022). This may have resulted in emotional distress and disordered eating in adolescents; however, no studies have examined the association between loneliness and breakfast skipping. Skipping breakfast is an unhealthy behaviour pattern that can transition from adolescence to adulthood with detrimental impacts on health (Smith et al., 2010). Many adolescents skip breakfast despite the benefits of regular breakfast consumption. In a nationally representative sample of Canadian adolescents, nearly 48.5% skipped breakfast at least once a week (Lazzeri et al., 2016). Loneliness, when included as a covariate, has been associated with lower odds of breakfast consumption in adolescents (Mathew et al., 2022).

Additionally, various lockdown measures imposed during the COVID-19 pandemic to limit the spread of the virus may have negatively impacted 24-hour movement behaviours. The 24-hour movement guidelines suggest ≥ 60 min/day of moderate-to-vigorous physical activity [MVPA], ≤2 h/day of recreational screen time [ST], and 8–10 h/night of sleep for adolescents (Tremblay et al., 2016). A scoping review exploring the impact of COVID-19 on movement behaviours in children and adolescents reported a consistent decline in MVPA, significant increases in ST and sleep duration, and a decrease in sleep quality (Paterson et al., 2021).

Two cross-sectional studies in adolescents found that youth who were less physically active had higher loneliness (Page et al., 2003, Pinto et al., 2021). However, in another study of adolescents aged 14–19 years, being physically inactive was not associated with feelings of loneliness (dos Santos et al., 2015). Loneliness in adolescents assessed prior to the COVID-19 pandemic has been associated with increased ST in cross-sectional analyses (Lawrence et al., 2022, MacDonald et al., 2022); however, this relationship was not supported when analysed longitudinally (MacDonald et al., 2022). Regarding loneliness and sleep duration, higher levels of loneliness were associated with inadequate sleep at night in adolescents (Eccles et al., 2020a). Finally, in a longitudinal study that examined different trajectories of loneliness in children and adolescents, no significant differences in sleep duration amongst the various loneliness groups were found (Eccles et al., 2020b). However, these studies analysed data collected prior to the COVID-19 pandemic.

With limited and contradicting pre-pandemic evidence in adolescents, the objective of the present study was to examine the association between loneliness levels due to the COVID-19 pandemic with eating habits and 24-hour movement behaviours. We hypothesized that increased loneliness in adolescents due to COVID-19 would result in skipping breakfast and lower likelihood of meeting the MVPA, screen time and sleep duration recommendations. As loneliness is known to steadily increase across the lifespan with chronic detrimental health implications, our study is the first to examine loneliness levels specifically due to the COVID-19 pandemic and its association with breakfast skipping and adherence to the 24-hour movement guidelines amongst a sample of Canadian adolescents. This information is important to inform future intervention strategies and public health policies.

## 2 Materials and methods

We used data from the 2020-21 wave of the COMPASS study (Cannabis, Obesity, Mental health, Physical activity, Alcohol, Smoking, and Sedentary behaviour), an ongoing prospective study, collecting annual health survey data from students in grades 9–12 (ages 12–19 years) attending participating secondary schools in Ontario, Alberta, British Columbia, and Quebec (secondary I-V), Canada (Leatherdale et al., 2014). All study protocols have been approved by the Human Research Ethics Board at the University of Waterloo (ORE #30118) and appropriate school board committees. Detailed information regarding the COMPASS design and methodology is available on the study website (https://uwaterloo.ca/compass-system/) and in print (Leatherdale et al., 2014). This paper used a cross-sectional study design and post-pandemic onset student data collected from November 2020 – June 2021.

We used adolescents’ self-reported loneliness levels (i.e., increased, decreased, or stayed the same) due to COVID-19 as our independent variable. We pooled “decreased” (n = 2,225) and “stayed the same” (n = 16,687) loneliness groups due to the small sample size in the “decreased only” group. Adolescents self-reported eating habits and 24-hour movement behaviours were used as outcomes. Variables were dichotomized (yes/no) for meeting the MVPA (≥60 min/day), recreational ST (≤2h/day) and sleep duration (≥8h/night) guideline recommendations. For eating habits, adolescents were asked to report if they had breakfast everyday (yes/no). For MVPA, adolescents were asked to report the number of minutes they spent doing moderate (i.e., low intensity workouts such as walking, biking to school and recreational swimming) and hard physical activity (i.e., jogging, team sports, fast dancing, jump-rope, or other activities that increased heart rate) during the past week. Regarding ST, adolescents were asked to report the number of hours per day they spent doing the following activities in the past week: a) watching/streaming TV shows or movies; b) playing video games/computer games; c) surfing the internet; and d) texting, messaging, and emailing. For sleep duration, adolescents were asked to report at what time they turned out the lights and went to sleep during the past week on weekdays and weekends, and at what time they woke up on weekdays and weekends. Additional information regarding the calculation of total MVPA, ST and sleep duration can be found in the notes under Table 1.

**Table 1:** Prevalence of eating habits, 24-hour movement behaviours, and covariates among Canadian adolescents by loneliness levels due to the COVID-19 pandemic, COMPASS study data 2020–21 (n = 40,521).

**Characteristics**	**Boys (n=18,294)**		**Girls (n=22,227)**	
	**Loneliness increased**	**Loneliness decreased/stayed the same**	**Loneliness increased**	**Loneliness decreased/stayed the same**
***Covariates***				
Age, years (mean, SD) *	15.1 (1.47)	14.7 (1.56)	15.0 (1.48)	14.8 (1.56)
SES category (%) *				
High SES	64.7	67.5	62.8	67.3
Low SES	35.3	32.5	37.2	32.7
Province (%) *				
Quebec	60.4	63.9	61.3	62.0
Ontario	26.8	23.1	26.6	23.1
British Columbia	8.10	8.80	8.03	10.7
Alberta	4.66	4.20	4.01	4.24
Ethnicity (%) *				
White	78.1	77.7	79.7	74.4
Non-white	21.9	22.3	20.3	25.6
Learning situation (%) *				
Online	49.9	44.7	51.5	44.8
In-person	27.8	33.9	26.0	34.6
Hybrid	22.3	21.5	22.5	20.6
Body weight categories (%) *				
Underweight/normal weight	75.3	75.5	83.5	84.2
Overweight/obesity	24.7	24.5	16.5	15.8

***Outcomes***				
**Eating habits**				
Ate breakfast everyday (%) *				
Yes	55.9	64.7	42.8	54.8
No	44.1	35.3	57.2	45.2
**24-hour movement behaviours**				
≥60 minutes of MVPA per day (%) *				
Yes	66.0	68.4	52.9	54.9
No	34.0	31.6	47.1	45.1
≤2 hours of screen time per day (%) *				
Yes	3.60	5.40	5.0	8.8
No	96.4	94.6	95.0	91.2
≥8 hours of sleep per night (%) *				
Yes	70.0	77.1	72.4	77.6
No	30.0	22.9	27.6	22.4

Note: Data for 41,927 students were available for loneliness levels due to the COVID-19 pandemic and the outcome variables. We have presented data for 40,521 students (18,294 boys and 22,227 girls) and excluded those in the “other” category for gender due to a small size (accounts for 3% of the total sample). Data are presented as mean (SD) for continuous variables and as percentages (%) for categorical data.

Missing data for covariates are: age (0.07%), SES category (31.3%), gender (3.4%), ethnicity (0.3%), body weight category (43.4%) and learning situation (0.28%). Missing data for covariates were re-coded as an unknown category to retain all data points.

SES was evaluated by creating a sum SES score using six items: Income level (Less than median income level = 0, Greater than or equal to median income level = 1); Environment (Rural = 0, Medium Urban = 1, Large Urban = 2); “In your house, do you have your own bedroom?” (1 = Yes, 0 = No); “Do you sometimes go to bed hungry because there is not enough money to buy food?” (1 = No, 0 = Yes); “Would you say that you and your family are more or less comfortable financially than the average student in your class?” (0 = Less comfortable, 1 = As comfortable, 2 = More comfortable); “How true are the following statements about COVID-19 for you right now? I am worried about my family being able to pay bills and expenses” (1 = Neutral/I do not know/Mostly false/False), 0 = True/Mostly True). Scores ranged from 0 to 9, with higher scores indicating higher SES. SES category was created using the median value for SES score (i.e., ≥7: High SES, <7: Low SES).

Time spent in moderate physical activity (e.g., walking, biking to school) and vigorous physical activity (e.g., jogging, team sports, fast dancing) were collected and combined to calculate total time spent in MVPA. The total was averaged to reflect the number of minutes spent doing MVPA per day.

Screen time was assessed by asking adolescents how much time they spend doing the following activities – a) Watching/Streaming TV or movies; b) Playing video games; c) Surfing the internet; and d) Texting, messaging, and emailing. Total screen time per day was calculated by adding responses from questions a to d.

Sleep duration was assessed by asking adolescents at what time they went to sleep and woke up during the past week. An average was calculated for number of hours for sleep duration per night.

*p < 0.01 for the comparison between increased loneliness and decreased/stayed the same loneliness for both boys and girls.

Abbreviations: SD - standard deviation; SES - socioeconomic status; MVPA - moderate-to-vigorous physical activity.

Covariates included age (years), gender (boy/girl/other), race/ethnicity (White/Non-White), province (Quebec, Ontario, British Columbia, Alberta), body weight category (body mass index [BMI] categories of underweight, normal weight, overweight, and obesity using the World Health Organization growth curves), learning mode (in-person, online, hybrid/blended), and socioeconomic status (SES) category (a composite of six items; see in notes under Table 1). For gender, as we did not have a sufficient sample size (n = 1,334) in the “other” category, they were not included in our analysis.

We used chi-squared tests to estimate the bivariate associations for categorical variables and t-tests for continuous variables. We conducted multivariable logistic regression models for all the outcomes. We tested for interactions between gender and loneliness in our models. We also tested for changes in odds ratios with age. We conducted a sensitivity analysis by removing the “decreased” loneliness group. Data analyses were performed using SAS version 9.4. We followed STROBE guidelines (Strengthening the Reporting of Observational Studies in Epidemiology) for cross-sectional studies (https://www.equator-network.org/reporting-guidelines/strobe/).

## 3 Results

We had a total of 40,521 adolescents (18,294 boys and 22,227 girls) with data available for loneliness due to COVID-19 (Table 1). In the group of adolescents with increased loneliness, we found a higher prevalence of skipping breakfast and lower prevalence of meeting the guidelines for MVPA, ST and sleep duration in comparison to those in the decreased/stayed the same loneliness group. For girls and boys in the increased loneliness group, we found a higher prevalence of overweight/obesity, online learning, and lower SES compared to girls and boys in the decreased/stayed the same loneliness group.

As the interaction term between loneliness and gender was significant for breakfast skipping and meeting screen time guidelines, we stratified our logistic regression results by gender for all the outcomes. As ten observations were missing for age, we were left with a total sample size of 40,511 (18,290 boys and 22,221 girls) in our logistic regression models. After adjusting for covariates, adolescents with increased loneliness during the COVID-19 pandemic were more likely to skip breakfast [boys: OR 1.40 (95% CI: 1.32, 1.49) and girls: OR 1.62 (95% CI: 1.53, 1.71)], exceed ST guidelines [boys: OR 1.43 (95% CI: 1.24, 1.66) and girls: OR 1.72 (95% CI: 1.54, 1.92)], and report insufficient sleep duration [boys: OR 1.38 (95% CI: 1.28, 1.48) and girls: OR 1.36 (95% CI: 1.27, 1.45)] compared to those with decreased/stayed the same loneliness (Table 2). The odds of not meeting MVPA guidelines was significantly associated with increased loneliness for boys [OR 1.07 (95% CI: 1.00,1.14), however, these results lack clinical significance. In girls, the odds of not meeting MVPA guidelines was not significantly associated with increased loneliness. In a sensitivity analysis, we found higher odds of skipping breakfast and not meeting the 24-hour movement guidelines regardless of age (see Supplementary Tables). We also reported the odds ratios for adolescents with increased loneliness due to COVID-19 with covariates (see Supplementary Tables). We performed another sensitivity analysis by removing those in the “decreased” loneliness group, which accounted for 5% of the total sample size, but observed no change in the odds ratios (data not shown).

**Table 2:** Logistic regression model results in adolescents reporting increased loneliness due to COVID-19 and its associations with eating habits and 24-hour movement behaviours.

Outcomes	Boys (n = 18,290)	Girls (n = 22,221)
*Eating habits*		
Ate breakfast daily		
Yes	1.00 (reference)	1.00 (reference)
No	1.40 (1.32, 1.49)	1.62 (1.53, 1.71)

*24-hour movement behaviours*		
MVPA (≥60 mins per day)		
Yes	1.00 (reference)	1.00 (reference)
No	1.07 (1.00, 1.14)	1.06 (0.99, 1.12)

Screen time (≤2 h per day)		
Yes	1.00 (reference)	1.00 (reference)
No	1.43 (1.24, 1.66)	1.72 (1.54, 1.92)

Sleep (≥8 h per night)		
Yes	1.00 (reference)	1.00 (reference)
No	1.38 (1.28, 1.48)	1.36 (1.27, 1.45)

Abbreviations: MVPA – Moderate-to-vigorous physical activity, OR - Odds Ratio, CI - Confidence interval.

Note: “Decreased/stayed the same” loneliness was used as the reference category in the logistic regression models. All models were adjusted for age, ethnicity, province, body weight category, learning mode, and socioeconomic status (SES) category.

## 4 Discussion

### 4.1 Key findings

To our knowledge, this is the first study examining the relationship between loneliness due to COVID-19 with eating habits and 24-hour movement behaviours in adolescents. This cross-sectional study found that adolescents with increased loneliness were more likely to skip breakfast, exceed ST guidelines and report shorter sleep duration compared to those with decreased/stayed the same loneliness. The odds of skipping breakfast and not meeting ST guidelines were higher in girls compared to boys for skipping breakfast. In boys and girls with increased loneliness, the odds of insufficient MVPA levels were not clinically significant. As this is the first study to examine loneliness in adolescence due to COVID-19 with eating habits and 24-hour movement behaviours, we do not have studies to directly compare our results to.

### 4.2 Eating habits

In the increased loneliness group, 44.1% of boys and 57.2% of girls skipped breakfast (compared to 35.3% of boys and 45.2% of girls in the decreased/stayed the same loneliness group). We found statistically significant higher odds of skipping breakfast in both boys and girls. A recent review found an average increase of 83% in the number of hospital admissions due to eating disorders in the paediatric population during the pandemic (Devoe et al., 2023). It also suggested that feelings of loneliness may have contributed to the worsening of eating disorder symptoms (Devoe et al., 2023). In girls, higher loneliness levels at age 12 was associated with higher BMI z-scores at age 13 (Qualter et al., 2018), which may lead to breakfast skipping as a compensatory weight loss strategy (Cohen et al., 2003). Loneliness in adolescents may lead to a loss of appetite, which may further lead to skipping meals. However, longitudinal studies are needed to corroborate our findings and to better understand the mechanisms through which loneliness may lead to skipping breakfast in adolescents.

### 4.3 24-hour movement behaviours

#### 4.3.1 Physical activity

In our sample, 52.9% of girls and 66.0% of boys with increased loneliness met the recommended guidelines for MVPA (compared to 68.4% of boys and 54.9% of girls in the decreased/stayed the same loneliness group). We found that the odds of insufficient MVPA were small and not clinically meaningful for boys and girls in the increased loneliness group with reference to those in the decreased/stayed the same loneliness group. Previous pre-pandemic cross-sectional research that examined physical activity in association with loneliness as an outcome reported mixed findings in adolescents (dos Santos et al., 2015, Page et al., 2003, Pinto et al., 2021). Physical activity can be obtained through different means (e.g., active play, sports, physical education, active transportation) and it is reassuring to note that increased loneliness was not unfavourably associated with physical activity levels in this study.

#### 4.3.2 Screen time

Almost all adolescents with increased loneliness did not meet the ST guidelines (96.4% of boys and 95.0% of girls compared to 94.6% of boys and 91.2% of girls in the decreased/stayed the same loneliness group). The odds of not meeting the ST guidelines were statistically significant in both boys and girls. Girls tend to spend more time watching TV, communicating online and using social media compared to boys, while boys spend more time playing video games (Thomas et al., 2020). MacDonald et al. (2022) assessed loneliness in Canadian adolescents using data collected between 2017 and 18 and one year apart (2018–19). The study found that loneliness was significantly associated with higher ST (watching TV, playing video games, texting), with associations more pronounced in girls (MacDonald et al., 2022). A study by Lawrence et al. (2022) also found that higher isolation loneliness scores among adolescents were associated with increases in passive ST and gaming.

#### 4.3.3 Sleep duration

In our sample, 30.0% of boys and 27.6% of girls with increased loneliness did not meet the sleep duration guidelines (compared to 22.9% of boys and 22.4% of girls in the decreased/stayed the same loneliness group). We found that the odds of shorter sleep duration were statistically significant in adolescents with increased loneliness. A study of Danish adolescents (11–15 years old) prior to the COVID-19 pandemic found that a higher loneliness score was associated with lower odds of experiencing adequate sleep at night (Eccles et al., 2020a). The stronger associations observed in our study could be since we examined data during the pandemic, with adolescents reporting higher levels of loneliness and exceeding ST guidelines, which might have further impacted adolescents sleep duration at night. In a longitudinal study, increased social media use was associated with shorter sleep duration in adolescents (Sampasa-Kanyinga et al., 2018).

### 4.4 Limitations

The study has several limitations. First, this is a cross-sectional study design, which limits our ability to determine causality and temporality of the relationship between loneliness in adolescents with eating habits and 24-hour movement behaviours. Second, self-reported data might be subject to recall bias and social desirability. Finally, the psychometric properties of some questions are unknown (e.g., loneliness levels due to COVID-19, breakfast measure, sleep duration).

## 5 Conclusion

While skipping breakfast is a common phenomenon amongst adolescents, no studies examined the association with increased loneliness. Findings from this study show for the first time that increased loneliness in adolescents due to the COVID-19 pandemic was associated with breakfast skipping, higher ST levels, and shorter sleep duration. Efforts to reduce loneliness and interventions to establish healthy lifestyle behaviours during adolescence are critical to preventing detrimental mental and physical health consequences across the lifespan. Future longitudinal studies in adolescents with gender-stratified results and objective measures are needed to confirm our findings.

## Funding

The COMPASS study has been supported by a bridge grant from the CIHR Institute of Nutrition, Metabolism and Diabetes (INMD) through the “Obesity – Interventions to Prevent or Treat” priority funding awards (OOP-110788; awarded to SL), an operating grant from the CIHR Institute of Population and Public Health (IPPH) (MOP-114875; awarded to SL), a CIHR project grant (PJT-148562; awarded to SL), a CIHR bridge grant (PJT-149092; awarded to KP/SL), a CIHR project grant (PJT-159693; awarded to KP), and by a research funding arrangement with Health Canada (#1617-HQ-000012; contract awarded to SL), a CIHR-Canadian Centre on Substance Abuse (CCSA) team grant (OF7 B1-PCPEGT 410–10-9633; awarded to SL), and a SickKids Foundation New Investigator Grant, in partnership with CIHR Institute of Human Development, Child and Youth Health (IHDCYH) (Grant No. NI21-1193; awarded to KAP) funds a mixed methods study examining the impact of the COVID-19 pandemic on youth mental health, and a CIHR Operating grant (UIP 178846; awarded to KP) funds a study examining the impact of the COVId-19 pandemic on youth health behaviours, leveraging COMPASS study data. The COMPASS-Quebec project additionally benefits from funding from the Ministère de la Santé et des Services sociaux of the province of Québec, and the Direction régionale de santé publique du CIUSSS de la Capitale-Nationale.

## CRediT authorship contribution statement

**Saniya Tandon:** Conceptualization, Data curation, Formal analysis, Writing – original draft, Writing – review & editing. **Karen A. Patte:** Writing – review & editing, Funding acquisition. **Gary S. Goldfield:** Writing – review & editing. **Scott T. Leatherdale:** Writing – review & editing, Funding acquisition. **Jean-Philippe Chaput:** Conceptualization, Writing – review & editing, Supervision.

## Declaration of Competing Interest

The authors declare that they have no known competing financial interests or personal relationships that could have appeared to influence the work reported in this paper.

## Data Availability

Data will be made available on request.
