# Supplementary material for: Loneliness during COVID-19 and its association with eating habits and 24-hour movement behaviours in a sample of Canadian adolescents
Source: Prev Med Rep. 2023 Jun 16;35:102287. doi: 10.1016/j.pmedr.2023.102287 (PMC10273770; doi:10.1016/j.pmedr.2023.102287)
Supplement: Supplementary data 1 [file mmc1.docx]

**Supplementary Table 1.** Logistic regression model results for adolescents with increased loneliness due to COVID-19 with covariates for eating habits.

| **Covariates** | **Boys (n=18,290)** | **Girls (n=22,221)** |
| --- | --- | --- |
| **Age, years** |  | |
| 12 | 1.45 (1.26, 1.66) | 1.97 (1.75, 2.22) |
| 12-13 | 1.66 (1.48, 1.87) | 2.01 (1.80, 2.24) |
| 13-14 | 1.63 (1.49, 1.77) | 1.87 (1.73, 2.03) |
| 14-15 | 1.59 (1.50, 1.70) | 1.75 (1.64, 1.86) |
| 15-16 | 1.56 (1.47, 1.67) | 1.64 (1.54, 1.72) |
| 16-17 | 1.54 (1.40, 1.68) | 1.53 (1.41, 1.66) |
| 17-18 | 1.51 (1.34, 1.71) | 1.43 (1.28, 1.59) |
| 18-19 | 1.48 (1.26, 1.73) | 1.33 (1.16, 1.54) |
| **SES category** |  | |
| High SES | 1.00 (reference) | 1.00 (reference) |
| Low SES | 1.46 (1.28, 1.65) | 1.60 (1.43, 1.80) |
| **Ethnicity** |  | |
| White | 1.00 (reference) | 1.00 (reference) |
| Non-white | 1.35 (1.18, 1.53) | 1.62 (1.44, 1.82) |
| **Province** |  | |
| Quebec | 1.00 (reference) | 1.00 (reference) |
| Ontario | 1.49 (1.33, 1.68) | 1.53 (1.37, 1.72) |
| British Columbia | 1.27 (1.03, 1.56) | 1.56 (1.31, 1.87) |
| Alberta | 1.16 (0.87, 1.53) | 1.38 (1.04, 1.83) |
| **Learning situation** |  | |
| In-person | 1.00 (reference) | 1.00 (reference) |
| Online | 1.42 (1.30, 1.55) | 1.52 (1.40, 1.65) |
| Hybrid | 1.28 (1.12, 1.45) | 1.64 (1.46, 1.85) |
| **Body weight category** |  | |
| Underweight/Normal weight | 1.00 (reference) | 1.00 (reference) |
| Overweight/Obesity | 1.40 (1.20, 1.63) | 1.46 (1.21, 1.76) |

**Note:** “Decreased/stayed the same” loneliness was used as the reference category in the logistic regression models. Skipping breakfast daily modelled as event.

**Supplementary Table 2.** Logistic regression model results for adolescents with increased loneliness due to COVID-19 with covariates for moderate-to-vigorous physical activity (MVPA).

| **Covariates** | **Boys (n=18,290)** | **Girls (n=22,221)** |
| --- | --- | --- |
| **Age** |  | |
| 12 | 1.12 (0.97, 1.28) | 1.09 (0.96, 1.22) |
| 12-13 | 1.23 (1.09, 1.39) | 1.29 (1.15, 1.43) |
| 13-14 | 1.21 (1.11, 1.32) | 1.26 (1.16, 1.37) |
| 14-15 | 1.19 (1.11, 1.27) | 1.24 (1.17, 1.32) |
| 15-16 | 1.17 (1.09, 1.25) | 1.22 (1.15, 1.30) |
| 16-17 | 1.15 (1.05, 1.26) | 1.20 (1.11, 1.30) |
| 17-18 | 1.13 (0.99, 1.28) | 1.18 (1.06, 1.31) |
| 18-19 | 1.11 (0.94, 1.30) | 1.16 (1.00, 1.34) |
| **SES category** |  | |
| High SES | 1.00 (reference) | 1.00 (reference) |
| Low SES | 1.10 (0.96, 1.25) | 1.07 (0.95, 1.20) |
| **Ethnicity** |  | |
| White | 1.00 (reference) | 1.00 (reference) |
| Non-white | 1.04 (0.91, 1.19) | 0.96 (0.86, 1.08) |
| **Province** |  | |
| Quebec | 1.00 (reference) | 1.00 (reference) |
| Ontario | 1.09 (0.96, 1.23) | 0.98 (0.87, 1.10) |
| British Columbia | 0.92 (0.74, 1.15) | 0.96 (0.79, 1.15) |
| Alberta | 0.95 (0.70, 1.29) | 1.12 (0.84, 1.48) |
| **Learning situation** |  | |
| In-person | 1.00 (reference) | 1.00 (reference) |
| Online | 1.12 (1.02, 1.23) | 1.05 (0.97, 1.14) |
| Hybrid | 1.04 (0.91, 1.19) | 1.15 (1.02, 1.30) |
| **Body weight category** |  | |
| Underweight/Normal weight | 1.00 (reference) | 1.00 (reference) |
| Overweight/Obesity | 1.10 (0.93, 1.29) | 1.04 (0.86, 1.25) |

**Note:** “Decreased/stayed the same” loneliness was used as the reference category in the logistic regression models. Not meeting MVPA guidelines modelled as event.

**Supplementary Table 3.** Logistic regression model results for adolescents with increased loneliness due to COVID-19 with covariates for sleep duration.

| **Covariates** | **Boys (n=18,290)** | **Girls (n=22,221)** |
| --- | --- | --- |
| **Age** |  | |
| 12 | 1.74 (1.46, 2.07) | 1.68 (1.44, 1.97) |
| 12-13 | 2.25 (1.93, 2.62) | 1.85 (1.59, 2.14) |
| 13-14 | 2.08 (1.86, 2.34) | 1.74 (1.56, 1.93) |
| 14-15 | 1.93 (1.78, 2.09) | 1.63 (1.51, 1.77) |
| 15-16 | 1.78 (1.65, 1.93) | 1.53 (1.43, 1.65) |
| 16-17 | 1.65 (1.51, 1.82) | 1.44 (1.32, 1.57) |
| 17-18 | 1.53 (1.34, 1.75) | 1.35 (1.20, 1.53) |
| 18-19 | 1.42 (1.19, 1.69) | 1.27 (1.08, 1.49) |
| **SES category** |  | |
| High SES | 1.00 (reference) | 1.00 (reference) |
| Low SES | 1.42 (1.22, 1.65) | 1.48 (1.28, 1.70) |
| **Ethnicity** |  | |
| White | 1.00 (reference | 1.00 (reference) |
| Non-white | 1.32 (1.16, 1.52) | 1.43 (1.26, 1.62) |
| **Province** |  | |
| Quebec | 1.00 (reference) | 1.00 (reference) |
| Ontario | 1.39 (1.23, 1.58) | 1.40 (1.24, 1.57) |
| British Columbia | 1.33 (1.07, 1.65) | 1.31 (1.10, 1.58) |
| Alberta | 1.10 (0.82, 1.46) | 1.02 (0.78, 1.34) |
| **Learning situation** |  | |
| In-person | 1.00 (reference) | 1.00 (reference) |
| Online | 1.42 (1.27, 1.59) | 1.45 (1.30, 1.62) |
| Hybrid | 1.25 (1.09, 1.44) | 1.27 (1.11, 1.45) |
| **Body weight category** |  | |
| Underweight/Normal weight | 1.00 (reference) | 1.00 (reference) |
| Overweight/Obesity | 1.28 (1.08, 1.51) | 1.35 (1.08, 1.67) |

**Note:** “Decreased/stayed the same” loneliness was used as the reference category in the logistic regression models. Not meeting sleep duration modelled as event.

**Supplementary Table 4.** Logistic regression model results for adolescents with increased loneliness due to COVID-19 with covariates for screen time.

| **Covariates** | **Boys (n=18,290)** | **Girls (n=22,221)** |
| --- | --- | --- |
| **Age** |  | |
| 12 | 1.37 (1.02, 1.84) | 1.94 (1.56, 2.41) |
| 12-13 | 1.61 (1.26, 2.05) | 2.20 (1.82, 2.66) |
| 13-14 | 1.63 (1.36, 1.95) | 2.11 (1.83, 2.42) |
| 14-15 | 1.65 (1.42, 1.92) | 2.02 (1.81, 2.46) |
| 15-16 | 1.67 (1.39, 2.01) | 1.94 (1.70, 2.20) |
| 16-17 | 1.69 (1.32, 2.17) | 1.85 (1.55, 2.21) |
| 17-18 | 1.72 (1.24, 2.39) | 1.78 (1.41, 2.24) |
| 18-19 | 1.74 (1.14, 2.65) | 1.70 (1.26, 2.29) |
| **SES category** |  | |
| High SES | 1.00 (reference) | 1.00 (reference) |
| Low SES | 1.42 (1.05, 1.92) | 1.61 (1.28, 2.01) |
| **Ethnicity** |  | |
| White | 1.00 (reference) | 1.00 (reference) |
| Non-white | 1.43 (1.05, 1.94) | 1.55 (1.22, 1.97) |
| **Province** |  | |
| Quebec | 1.00 (reference) | 1.00 (reference) |
| Ontario | 1.61 (1.18, 2.20) | 2.18 (1.73, 2.76) |
| British Columbia | 1.09 (0.67, 1.75) | 1.39 (0.97, 1.97) |
| Alberta | 1.03 (0.49, 2.18) | 1.44 (0.84, 1.78) |
| **Learning situation** |  | |
| In-person | 1.00 (reference) | 1.00 (reference) |
| Online | 1.31 (1.06, 1.62) | 1.69 (1.44, 1.98) |
| Hybrid | 1.87 (1.30, 2.69) | 1.73 (1.36, 2.20) |
| **Body weight category** |  | |
| Underweight/Normal weight | 1.00 (reference) | 1.00 (reference) |
| Overweight/Obesity | 1.17 (0.78, 1.75) | 1.65 (1.07, 2.54) |

**Note:** “Decreased/stayed the same” loneliness was used as the reference category in the logistic regression models. Not meeting screen time guidelines modeled as event.
